# Practical Departments

**Published:** 1901-06-01

**Authors:** 


### PRACTICAL DEPARTMENTS.

### TILES FOR HOSPITAL FLOORS.

(MESSRS. PERCY BACON & BROTHERS, 11 NEWMAN STREET, OXFORD STREET, W., with whom Messrs. CARTER JOHNSON & CO. have amalgamated their interests.)

WE have lately visited the studios at 11 Newman Street, Oxford Street, where stained-glass windows, wrought-iron work, wood and stone carving, &c., are made. We have seen there a large variety of neat plain and glazed tiles, as well as antique encaustic tiles, and as this firm has made the production of these a distinct study, intending purchasers or hospital committees would do wisely to visit the Newman Street Studios before making their decision as to floors. Messrs. Bacon have many original designs for dados and ornamental wall tiling; and as the whole process, from designing in the studios to the last stage of fixing, is carried out on the spot by experienced workmen, orders can be executed at the shortest notice consistent with first-rate workmanship. The long list of architects for whom work has been done speaks for itself, and many well-known hospitals have been supplied by this firm.

For durability, as well as for sanitary reasons, Messrs. Bacon strongly recommend the interlocking rubber tiling made by the B. & S. Folding Gate Co., which they are prepared to supply on advantageous terms. It may be seen in use at their studios. It is noiseless, non-slippery, waterproof, thoroughly sanitary and of extraordinary durability. It has been in use on the main stairway landing of Broad Street Station, Penn. R.R. Co., Philadelphia, for six years, and is perfectly good to-day. Stains wash off completely, leaving the tile as bright and clean as when first laid. Two hundred and twenty-five blocks are required to cover one square yard; each tile is 2⅝ inches square and ⅝ of an inch thick, and it will be seen that the principle is that known as dovetailing. The interlocking feature unites the tiles into a smooth unbroken sheet of rubber; the tiles do not pull apart or come up, and as each is distinct, any desired colour scheme can be carried out. It can, moreover, be laid by an ordinary mechanic, and may be put down directly over the original floor of wood, stone, concrete, or metal. Jarring and wrenching have no effect on it, so that for lifts, for example, it is the most durable floor that can be required.

